# New algorithm for management of multiligament knee injuries: A tertiary level trauma center experience

**DOI:** 10.1002/jeo2.70387

**Published:** 2025-07-27

**Authors:** Ayman AbdelKawi, Mohammed Fargaly, Gaber Eid, Maher El Assal, Hesham Elkady, Tarek N. Fetih

**Affiliations:** ^1^ Orthopaedics and Traumatology Assiut University Hospital Assuit University Assuit Egypt; ^2^ Orthopedic Department Alazhar University, Assuit University Assuit Egypt

**Keywords:** clinical outcomes, knee dislocation, multiligament knee injury, return to work, Schenck's classification

## Abstract

**Purpose:**

Multiligament knee injuries (MLKIs) are severe orthopedic traumas frequently associated with concomitant structural damage, often leading to significant long‐term morbidity. This study aimed to evaluate the rate of return to work (RTW) following the management of MLKIs using a standardized treatment algorithm.

**Methods:**

This prospective interventional study included patients with MLKIs who presented to a tertiary trauma center between 2019 and 2022. A total of 32 patients (30 males and 2 females) were enrolled and classified according to the Schenck classification system. The median age at the time of injury was 31 years (range: 17–60 years). The mechanism of injury was high‐energy trauma in 21 patients, sports‐related trauma in eight patients, and low‐energy trauma in three patients. Clinical outcomes were assessed at final follow‐up using the Lysholm score, International Knee Documentation Committee (IKDC) subjective knee evaluation form, University of California Los Angeles (UCLA) activity score, and return to work status.

**Results:**

At a mean 2‐year postoperative follow‐up, the average range of motion across all patients was 134.2° ± 16.6°. The mean postoperative Lysholm, IKDC, and UCLA scores were 86.4 ± 12.6, 65.9 ± 9.7, and 6.9 ± 2.2, respectively. Notably, 90.6% of the patients achieved a successful return to their previous work. The proportion of patients returning to work was significantly higher in the Knee Dislocation Injury (KDI) group compared to the other three Schenck classification groups, which showed no significant difference among themselves. Radiographic evidence of osteoarthritis (OA) was observed in four cases (12.5%).

**Conclusion:**

This study demonstrates that satisfactory to excellent short‐term clinical outcomes, including a high rate of return to work, can be achieved following ligament reconstruction for multiligament knee injuries when utilizing a standardized treatment algorithm. However, the potential for long‐term complications, such as the development of knee osteoarthritis, warrants careful consideration and continued monitoring.

**Level of Evidence:**

Level IV.

**Abbreviations**
ACL: anterior cruciate ligament
BMI: body mass index
CPM: continuous passive motion
CT: computed tomography
FDS: fall down stairs
FOG: fall on ground
G: gracillis
H: hamstrings
IKDC: International Knee Documentation Committee
KD: knee dislocation
K‐L system: Kellgren–Lawrence Classification System
K‐wires: Kirschner wires
MBA: motor bike accident
MCA: motor car accident
MLKI: multiligament knee injuries
MRI: magnetic resonance imaging
ORIF: open reduction internal fixation
PCL: posterior cruciate ligament
PL: peroneus longus
PLC: posterolateral complex
Q: quadriceps
ROM: range of motion
RTW: return to work
SBT: split Biceps transfer
semiT: semitendinosis tendon
sMCL: superficial medial collateral ligament
SPSS: Statistical Package for the Social Sciences
TKA: total knee arthroplasty
UCLA: University of California Los Angeles

## INTRODUCTION

Multiligament knee injuries (MLKIs) are characterized by the disruption of two or more of the knee's primary ligaments [16]. Although relatively infrequent, these injuries are devastating, frequently accompanied by damage to adjacent structures, and often lead to significant long‐term morbidity. The initial priority in managing MLKIs is addressing any life‐ and limb‐threatening conditions. The presence of associated fractures and nerve injuries can further complicate the treatment course. In 1994, Schenck proposed the most widely adopted classification system for MLKIs, based on the anatomical pattern of the torn ligaments (Table 1) [20]. This classification is advantageous as it precisely defines the injured ligaments, thereby aiding in treatment planning. The recovery of knee function following MLKIs is primarily influenced by the stability and range of motion achieved in the knee joint, as well as patient age and body mass index (BMI) [9].

**Table 1:** Schenck's classification.

KD I	Injury to single cruciate + collaterals
KD II	Injury to ACL and PCL with intact collaterals
KD III	M Injury to ACL, PCL, MCL
KD III	L Injury to ACL, PCL, FCL
KD IV	Injury to ACL, PCL, MCL, FCL
KD V	Dislocation + fracture

*Note*: Additional caps of ‘C’ and ‘N’ are utilized for associated injuries. ‘C’ indicates an arterial injury. ‘N’ indicates a neural injury, such as the tibial or, more commonly, the peroneal nerve.

Abbreviations: ACL, anterior cruciate ligament; FCL, fibular collateral ligament; KD, Knee Dislocation Classification I–V; MCL, medial collateral ligament.

The management of these complex knee injuries remains a subject of debate. There is a lack of definitive consensus regarding the optimal approach, including the use of staged versus single‐stage surgical procedures, ligament repair versus reconstruction, the specific type of reconstruction for each damaged ligament, the optimal timing of surgery, graft selection, the sequence of ligament reconstruction and postoperative rehabilitation protocols [14]. The variability in associated orthopedic and non‐orthopedic injuries results in heterogeneous injury patterns with numerous confounding variables, making controlled research trials challenging. Consequently, the highest level of available evidence often consists of descriptive, retrospective studies with a low level of evidence [10].

The purpose of this study was to analyze the experience of our institution, a tertiary‐level trauma center, in managing multiligament knee injuries using a standardized treatment algorithm. This algorithm is guided by the specific types of injuries, the rationale for definitive treatment strategies, and structured rehabilitation protocols. We evaluated postoperative complications and functional outcomes, including the ability of patients to return to work.

## MATERIALS AND METHODS

Following approval from the institutional review board (IRB# 17200541), this prospective interventional study enrolled 43 consecutive patients with multiligament knee injuries who presented to Assiut University Hospitals between 2019 and 2022. Emergency surgical procedures were conducted in the trauma department, while elective surgeries were performed by one of sports medicine orthopedic surgeons within the Arthroscopy and Sport Injuries unit.

**Inclusion criteria:**

- Patients presenting to the emergency department of Assiut University Hospital in the acute stage with a confirmed knee dislocation.
- Patients presenting to the outpatient sports clinic with chronic injuries involving two or more knee ligaments.
- Age range of 17–60 years.
- Provision of informed consent by the patient.

**Exclusion criteria:**

- Pre‐existing advanced osteoarthritis in the affected knee.
- Injury to the contralateral knee that would interfere with graft harvesting.
- Current septic arthritis of the ipsilateral knee.
- Patients deemed unlikely to comply with the postoperative rehabilitation program.
- Patients with severe systemic medical conditions that would interfere with major surgical operations.
- Recent history of substance abuse (e.g., recreational drugs, alcohol) that would preclude compliance with the rehabilitation program and reliable assessment.

Based on the aforementioned criteria, 11 patients were excluded from the initial cohort of 43 patients who presented to our institution. Diagnoses were established through a comprehensive evaluation encompassing patient history, thorough clinical examination, and magnetic resonance imaging (MRI). The included patients were subsequently classified according to the Schenck classification system. All patients underwent a detailed clinical vascular and neurological assessment. For patients presenting with acute MLKIs complicated by vascular injury, polytrauma or gross knee instability following closed reduction, a cross‐knee external fixator was applied. The remaining patients with acute injuries were managed with a hinged knee brace. Limb malalignment in chronic cases was evaluated using a scanogram.

These fixators were removed within 2–6 weeks after the application. The delay in the removal of the external fixator was due to other major injuries and bad general conditions interfering with earlier removal.

**Figure 1:**
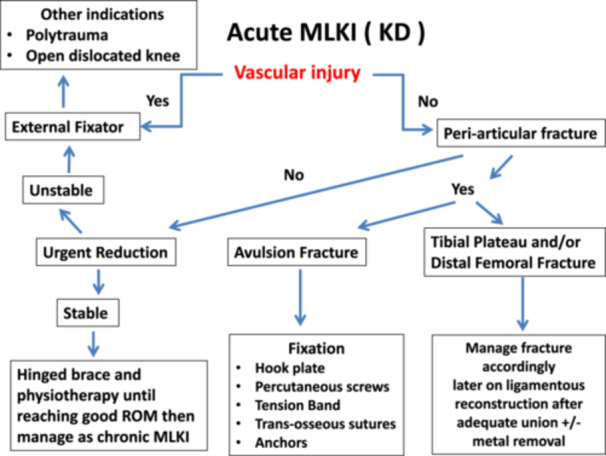
Protocol for management of acute multiligament knee injuries (MLKI). KD, knee dislocation.

**Figure 2:**
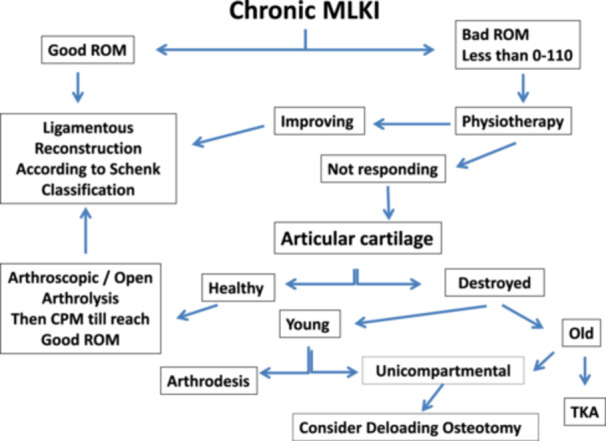
Protocol for management of chronic MLKI. CPM, continuous passive motion; MLKI, multiligament knee injuries; ROM, range of motion; TKA, total knee arthroplasty.

Seven patients presented with fractures around the knee (five tibial plateau fractures, one distal femur fracture and one combined tibial plateau and distal femur fracture). In these cases, open reduction and internal fixation (ORIF) of the fracture was performed. Ligamentous reconstruction was subsequently undertaken after complete bony union and removal of any osteosynthesis hardware that could interfere with the creation of bony tunnels required for ligament graft placement. The standardized protocol for the management of MLKIs is illustrated in Figures 1 and 2.

Preoperative Lysholm, International Knee Documentation Committee (IKDC), and University of California Los Angeles (UCLA) scores were documented for patients presenting with chronic MLKIs. In acute cases managed with staged surgical procedures, these scores were obtained prior to the second‐stage surgery. The average interval between the initial presentation and the definitive surgical intervention was 8 weeks, ranging from 6–12 weeks.

### Operative details of the ligamentous reconstruction surgeries

Definitive ligamentous reconstruction was performed once patients achieved a knee range of motion of at least 0° of extension to 120° of flexion. All ligamentous reconstructions in chronic cases were conducted in a single surgical session. Patients were positioned supine on a standard orthopedic operating table. In cases of knee dislocation (KD) types III and IV, the contralateral limb was prepared and draped to allow for free tendon graft harvesting. Examination under anesthesia was performed and meticulously documented for all patients. Fluoroscopic imaging was readily available to ensure accurate tunnel placement.

The operating surgeon commenced with a standard arthroscopic examination of the injured knee to definitively confirm the torn ligaments. Subsequently, the necessary tendon grafts were harvested. While the surgical assistant prepared these grafts for reconstruction on a separate sterile table, the surgeon addressed any meniscal pathology (either repair or partial meniscectomy based on the tear pattern), debrided any chondral lesions, removed intra‐articular loose bodies, excised any restrictive intra‐articular adhesions, and performed notchplasty to widen a narrow intercondylar notch, thereby minimizing the risk of future graft impingement and subsequent failure. Following these steps, the bony tunnels required for ligamentous reconstruction were meticulously prepared. Graft insertion and fixation were then performed. In two specific cases, corrective osteotomy was performed without concurrent ligamentous reconstruction; one following a malunited distal femoral fracture and the other after a malunited tibial plateau fracture.

### Graft harvesting

**Figure 3:**
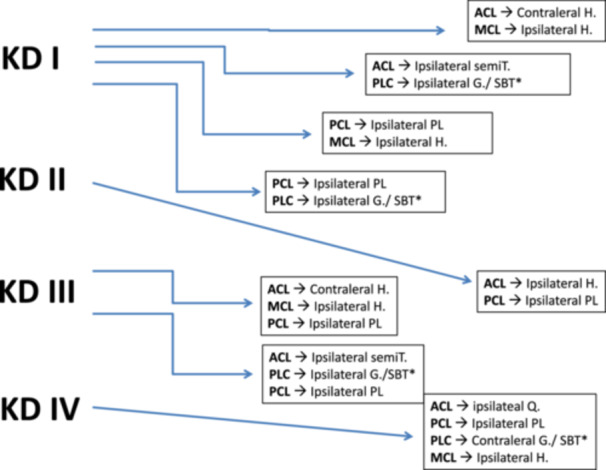
Graft selection in the chronic cases according to Schenck classification. ACL, anterior cruciate ligament; G, gracillis; H, hamstrings; KD, knee dislocation; MCL, medial collateral ligament; PL, peroneus longus tendon; PCL, posterior cruciate ligament; PLC, posterolateral complex; Q, quadriceps tendon; semiT, semitendinosis tendon. *SBT, split biceps transfer done if the head of fibula was very porotic, very small or had an old healed fracture.

Due to the limited availability of allografts in our region, autografts were utilized exclusively for all ligament reconstructions. In the majority of cases requiring anterior cruciate ligament (ACL) reconstruction, ipsilateral or contralateral hamstring tendons were harvested. However, in type KD IV injuries, an ipsilateral quadriceps tendon graft was preferred. This decision was dictated by the use of both ipsilateral hamstring tendons for superficial medial collateral ligament (sMCL) reconstruction and the contralateral gracilis tendon for posterolateral corner (PLC) reconstruction utilizing a modified Larson technique. In instances where the fibular head exhibited significant osteoporosis, was notably small, or presented with evidence of a previously healed fracture, PLC augmentation was performed using a split biceps femoris tendon transfer. For all posterior cruciate ligament (PCL) reconstructions, the ipsilateral peroneus longus tendon was consistently selected as the graft source. Figure 3 provides a detailed overview of the graft selection strategy employed based on the Schenck classification of multiligament knee injuries.

### Rehabilitation

Rehabilitation following multiligament knee reconstruction presents significant challenges due to the diverse injury patterns and the extensive damage sustained by the soft tissue structures of the knee. The primary objectives during the early postoperative period include safeguarding the reconstructed ligaments through appropriate bracing and weight‐bearing restrictions, managing pain and swelling, initiating early range of motion (ROM) exercises, promoting quadriceps muscle activation, and providing comprehensive patient education regarding activity precautions and expected recovery timelines. Patients were maintained on a strict non‐weight‐bearing protocol for the initial 6 weeks postoperatively, with the knee immobilized to ensure joint stability. Patients who underwent posterior cruciate ligament reconstruction (PCLR) were transitioned to a dynamic PCL brace to provide ongoing support to the healing grafts as soon as postoperative swelling subsided sufficiently to ensure proper brace fit. In cases involving reconstruction of one or both collateral ligaments, a hinged knee brace was applied for a duration of four months to mitigate varus and valgus stresses on the knee. Gradual progression of range of motion exercises, along with gait retraining, was initiated after the initial 6‐week period. Return to sports activities and manual labor was typically permitted within a timeframe of 9–12 months postoperatively.

### Statistical methods

Data management and statistical analysis were performed using IBM SPSS Statistics for Windows, Version 27.0 (Armonk, NY: IBM Corp.). The chi‐square test or Fisher's exact test was employed to assess the independence of proportions between Schenck's classification and patient demographic characteristics, BMI, mechanism of trauma, radiological signs of injury, management modalities, and postoperative complications. Non‐parametric paired t‐tests were utilized to compare clinical and functional outcome parameters before and after surgical management, stratified by Schenck's classification. A *p*‐value of ≤ 0.05 was considered statistically significant for all analyses.

## RESULTS

Thirty‐two patients were included in this study and evaluated at a minimum follow‐up of two years. Twelve patients sustained injury to the right knee, while twenty sustained injury to the left knee. The median age at the time of surgery was 31 years, with an age range of 17–60 years. The study cohort comprised two female and 30 male participants. The majority of patients were classified as non‐obese (BMI < 25 kg/m²). One patient was morbidly obese (BMI 40 kg/m²), and eight patients were obese, with a median BMI of 35 kg/m² among this subgroup. Regarding occupation, six patients were manual laborers, eight were farmers, and the remaining patients were involved in light work. The cohort included one patient with diabetes mellitus, three patients with hypertension, and five patients who were active smokers.

According to the mechanism of injury, twenty‐one patients sustained high‐energy trauma, including motor vehicle accidents (*N* = 10), motorbike accidents (*N* = 9) and falls from height (*N* = 2). Eight cases resulted from sports‐related trauma, and three cases were attributed to low‐energy trauma (two falls down stairs involving fewer than six steps and one fall on the ground [FOG]). Table 2 provides a detailed breakdown of the injury mechanisms observed in this cohort.

**Table 2:** Mechanism of injury of the cases.

	KD I		KD II		KD III		KD IV		Total	
	No	%	No	*%*	No	No	%	No	NO	%
FDS	2	10.6	–	–	–	–	–	–	2	6.2
FFH	1	5.3	–	–	1	16.7	–	–	2	6.3
FOG	–	–	–	–	1	16.7	–	–	1	3.1
MBA	7	36.8	–	–	1	16.7	1	25.0	9	28.1
MCA	3	15.8	1	33.3	3	50.0	3	75.0	10	31.3
Sport knee trauma	6	31.6	2	66.7	–	–	–	–	8	25.0

Abbreviations: FDS, fall down stairs; FFH, falls from height; FOG, fall on ground; KD, knee dislocation; MBA, motor bike accident; MCA, motor car accident.

Regarding the specific ligaments injured, Table 3 summarizes the distribution of cases according to the Schenck classification system.

**Table 3:** Injury pattern according to Schenck classification.

Ligamentous injury	KD I	KD II	KD III	KD IV	Total
ACL, MCL	4	–	–	–	4
ACL, PLC	5	–	–	–	5
PCL, MCL	2	–	–	–	2
PCL, PLC	7	–	–	–	7
PCL, PLC, MCL	1	–	–	–	1
ACL, PCL.	–	3	–	–	3
ACL, PCL, MCL.	–	–	2	–	2
ACL, PCL, PLC	–	–	4	–	4
ACL, PCL, PLC, MCL	–	–	–	4	4
Total	19 (59.3%)	3 (9.3%)	6 (18.7%)	4 (12.5%)	32

Abbreviations: ACL, anterior cruciate ligament; MCL, medial collateral ligament; PCL, posterior cruciate ligament; PLC, posterolateral corner.

Regarding meniscal tears, the management strategy was determined by the tear type, location, and the chronicity of the injury (time elapsed between the initial trauma and the definitive surgical intervention). Meniscal tears were identified in 14 cases: three cases underwent trimming of combined medial and lateral meniscal tears, one case had lateral meniscal tear trimming, six cases had medial meniscal tear trimming, two cases underwent all‐inside repair of medial meniscal tears, and two cases had inside‐out repair of medial meniscal tears. Additionally, five patients exhibited ulceration of the articular surface of the medial femoral condyle, for which arthroscopic microfracture was performed.

At the final follow‐up, with a mean of 2 years postoperatively, the average range of motion across all cases was 134.2° ± 16.6°. The mean preoperative Lysholm, IKDC and UCLA scores, obtained before the definitive surgical intervention, were 70 ± 8.6, 53.7 ± 7.0 and 4.0 ± 1.1, respectively. At the 2‐year postoperative assessment, the mean Lysholm, IKDC, and UCLA scores were 86.4 ± 12.6, 65.9 ± 9.7 and 6.9 ± 2.2, respectively. Statistical analysis revealed a significant improvement between the preoperative and 2‐year postoperative scores across all cases and specifically within the KD type I and type III subgroups (*p* < 0.05). Table 4 provides a detailed overview of the functional outcomes within each Schenck classification group.

**Table 4:** Distribution and comparison of clinical and functional outcome parameters before and after management sorted by Schenck's classification.

Study group	Lysholm preop.	Lysholm postop.	*p* value	IKDC preop.	IKDC postop.	*p* value	UCLA preop.	UCLA postop.	*p* value	Active ROM postop.
KD I	72.4 ± 6.8	89.7 ± 8.8	<**0.001**	56.2 ± 6.2	68.5 ± 8.2	<**0.001**	4.6 ± 1.0	8.1 ± 14	<**0.001**	142.4 ± 8.1
*N* = 19										
KD II	65.0 ± 8.6	75.7 ± 13.6	0.109	53.3 ± 5.8	61.0 ± 8.7	0.109	3.7 ± 1.2	5.3 ± 3.2	0.180	116.7 ± 30.6
*N* = 3										
KD III	70.2 ± 4.1	85.5 ± 10.1	**0.028**	50.5 ± 6.1	64.3 ± 13.0	**0.028**	3.0 ± 0.0	5.7 ± 2.2	**0.043**	130.0 ± 17.9
*N* = 6										
KD IV	62.5 ± 16.6	79.8 ± 25.5	0.066	47.0 ± 8.3	59.8 ± 10.3	0.068	3.0 ± 0.8	4.5 ± 1.7	0.109	115.0 ± 5.8
*N* = 4										
Total	70.0 ± 8.6	86.4 ± 12.6	<**0.001**	53.7 ± 7.0	65.9 ± 9.7	<**0.001**	4.0 ± 1.1	6.9 ± 2.2	<**0.001**	134.2 ± 16.6
*N* = 32										

Abbreviations: IKDC, International Knee Documentation Committee; KD, knee dislocation; ROM, range of motion; UCLA, University of California Los Angeles.

**Figure 4:**
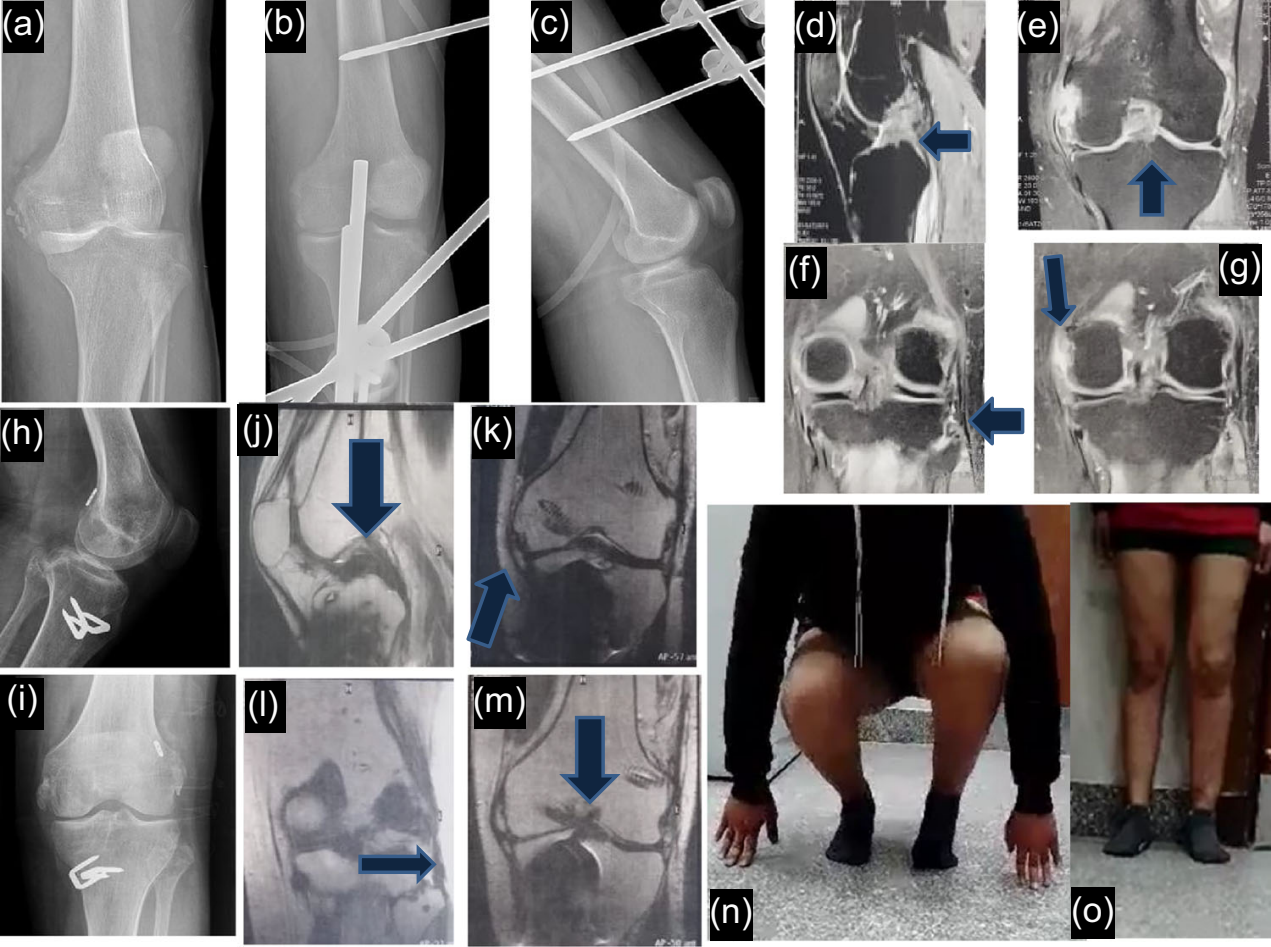
(a) Preoperative x‐ray AP view of the dislocated knee. (b & c) Postoperative x‐ray AP and Lateral views of knee after reduction with cross knee external fixator. (d–g) Preoperative fat‐suppressed coronal and sagittal MRI images showing PCL, ACL, PLC and MCL injury. (h & i) Two years postoperative follow up x‐ray AP and lateral views of the knee showing PCL, ACL and PLC reconstruction. (j–m): Two years postoperative follow‐up MRI knee showing PCL, ACL, MCL and PLC reconstruction. (n & o): Two years postoperative clinical photo of the knee range of motion. ACL, anterior cruciate ligament; MCL, medial collateral ligament; PCL, posterior cruciate ligament; PLC, posterolateral corner

Notably, 90.6% of the patients achieved a successful return to their previous work. The proportion of patients returning to work was significantly higher in the KD type I group compared to the other three Schenck classification groups (KD types II, III and IV), which did not demonstrate significant differences among themselves. Table 5 illustrates the return to work rates according to the Schenck classification. Figure 4 depict the management of a patient who sustained a type IV KD injury and successfully returned to work.

**Table 5:** Return to work according to Schenck classification.

		Schenck classification										*p* value
		KD I		KD II		KD III		KD IV		Total		
		Count	%	Count	%	Count	%	Count	%	Count	%	
Return to work	Y	19	100.0%	2	66.7%	5	83.3%	3	75.0%	29	90.6%	
	N	0	0.0%	1	33.3%	1	16.7%	1	25.0%	3	9.4%	0.069
	Total	19	100.0%	3	100.0%	6	100.0%	4	100.0%	32	100.0%	

Abbreviation: KD, knee dislocation.

Postoperative complications included arthrofibrosis in two cases. One patient with arthrofibrosis experienced improvement with a dedicated physiotherapy program, while the other required arthroscopic arthrolysis. A non‐displaced intra‐articular fissure fracture extending from a high tibial osteotomy occurred in one patient. Revision PCL reconstruction was performed in one patient who sustained a type I knee dislocation injury; this revision utilized a contralateral hamstring tendon graft. Radiographic signs of osteoarthritis, graded according to the Kellgren–Lawrence (K–L) system, developed in four cases (12.5%): one case with K–L grade 2 and three cases with K–L grade 3. When categorized by Schenck classification, these cases comprised one KD type II, one KD type III, and two KD type IV injuries. Patients exhibiting radiographic evidence of osteoarthritis were managed conservatively.

## DISCUSSION

In this study, the clinical outcomes of multiligament knee injuries were analyzed. This type of knee trauma presents numerous challenges and complexities that must be addressed during management [1, 4, 11, 21]. The primary finding of this study is that surgical treatment of MLKIs yields satisfactory results with respect to knee scores (Lysholm, IKDC and UCLA), range of motion, and return to work at a two‐year follow‐up. Klasan et al., in their recent systematic review and meta‐analysis of 3571 patients across 79 studies with MLKIs and a minimum 2‐year follow‐up, reported that patients who sustain an MLKI can expect to regain approximately 80‐85% of knee function at 2 years [13]. Ligamentous reconstruction is considered the gold standard treatment for MLKIs in the literature [3, 7, 19]. MLKIs are relatively rare injuries, accounting for 0.02%–0.2% of all orthopedic injuries, which explains the limited number of cases in published case series, a finding comparable to our work [18, 24].

In the present study, the majority of patients were male and had sustained high‐energy trauma. Previous reports indicate that MLKIs typically affect either young patients experiencing high‐energy trauma or older, obese patients suffering simple falls [15, 23]. Literature suggests that approximately one‐third of MLKI cases are associated with fractures [12]. Tardy et al., in their case series of 39 patients, reported 16 cases (41%) with meniscal tears, with 46% classified as KD type I. Similarly, Hantes et al. and Tardy et al. found 15 cases with KD type I, representing about 57.6% of their cohorts [8, 22]. Consistent with these findings, our study identified 59.3% of cases as type I KD, and 43.7% of patients presented with meniscal pathology that was addressed during ligamentous reconstruction.

Patients with multiligament knee injuries face a high risk of complications and long‐term disability. Several factors contribute to this increased risk, including the severity of the initial injury, pre‐existing conditions at the time of surgical reconstruction (limb malalignment, meniscal injury or cartilage pathology), failure to identify all knee injuries, technical errors (e.g., failure to reconstruct a lax ligament, use of a structurally weak graft, non‐anatomic tunnel placement or inadequate graft fixation), an insufficient rehabilitation protocol, and potential subsequent traumatic events. However, a systematic review by Emre et al. suggests that advanced age, early ligamentous reconstruction, and high‐energy trauma resulting in injury to three or more ligaments are associated with the poorest functional outcomes [6, 17]. This aligns with our results, where the primary complication observed was osteoarthritis (12.5%). Half of these patients were over 55 years old at the time of injury, and the other half had sustained injury to all four major knee ligaments. The development of posttraumatic knee osteoarthritis is significantly influenced by the severity of the initial trauma and the presence of associated injuries to the cartilage (chondral), menisci and bone. These factors were substantially observed in our patient cohort.

In the literature, the rate of return to pre‐injury activity level after knee dislocations and multiligament injuries varies. Everhart et al., in their systematic review of 524 patients, reported a return to work rate of 60% following surgical management of multiligament knee injuries, while D'Ambrosi et al. published a recent systematic review of 439 patients showing that 75% of patients returned to a pre‐injury activity level [2, 5]. In contrast, our study demonstrated a higher rate, with 90.6% of cases successfully returning to their pre‐injury activity level. This discrepancy is likely attributable to the significant proportion of our cohort with lower energy trauma (KD type I and II), which generally exhibit more favorable functional outcomes.

To achieve satisfactory results in the management of this complex knee trauma, a clear algorithm for both acute and chronic MLKI cases has been established at this institution. In acute cases, vascular injury represents the most critical associated injury, as delayed or inadequate treatment can lead to amputation. Therefore, urgent reduction of the dislocated knee with external fixation application and vascular exploration is paramount. In the absence of vascular injury, the priority shifts to addressing associated fractures, whether juxta‐articular (tibial plateau and/or distal femoral fracture) or avulsion fractures. Pure ligamentous knee injuries are initially managed with a hinged knee brace and subsequently treated after the reduction of swelling and the recovery of an acceptable range of motion, similar to chronic cases.

In chronic MLKI cases, regaining a satisfactory range of motion is the most crucial and often the most challenging objective. Failure to achieve adequate range due to extensive chondral injury may necessitate arthroplasty in older patients. Arthroscopic or open arthrolysis is the preferred approach to restore the desired range of motion for subsequent ligamentous reconstruction, provided the articular cartilage is sufficiently healthy.

The primary limitation of this study lies in the heterogeneity of the included patients concerning the severity of injury, the pattern of injured ligaments (bony avulsion, distal, proximal or mid‐substance injury), the timing of treatment, the specific treatment modality (repair or reconstruction), and the presence of other associated orthopedic and non‐orthopedic injuries. Another limitation of this study is that the surgical procedures were performed by different surgeons, potentially introducing variability in technique and outcomes. Moving forward, the aim is to establish a standardized, universally applicable algorithm for the management of this complex knee trauma. There is also an intention to categorize patients into clearer subgroups with larger numbers in each group to obtain more robust and reliable results in future investigations. However, a significant strength of this study, unlike the majority of existing literature on multiligament injured knees which are retrospective in nature, is its prospective design.

## CONCLUSION

Following the algorithm utilized, satisfactory to excellent short‐term clinical results, as evidenced by return to work, can be achieved through ligament reconstruction in patients with multiligament knee injuries. However, the potential for long‐term consequences, such as knee osteoarthritis, should be considered.

## AUTHOR CONTRIBUTIONS

Ayman AbdelKawi: Final approval of the version to be published. Mohammed Fargaly: Drafting the work, reviewing it critically for important intellectual content and final approval of the version to be published. Gaber Eid: Drafting the work and reviewing it critically for important intellectual content. Maher El Assal: Substantial contributions to the design of the work. Hesham Elkady: Substantial contributions to the design of the work. Tarek Fetih: Final approval of the version to be published.

## CONFLICT OF INTEREST STATEMENT

The authors declare no conflicts of interest.

## ETHICS STATEMENT

This research was approval by the Faculty Ethics Review Board, Assiut University (IRB local approval number 17200541).

## Data Availability

None declared.

## References

[1] Angelini FJ , Helito CP , Bonadio MB , da Mota E Albuquerque RF , Pecora JR , Camanho GL . Surgical management of knee dislocations with ligament reconstruction associated with a hinged external fixator. Orthop Traumatol Surg Res. 2015;101(1):77–81. 25530481 10.1016/j.otsr.2014.11.001

[2] D'Ambrosi R , Meena A , Ursino N , Di Feo F , Fusari N , Kambhampati SBS . Return to sport after multiligament knee injury: a systematic review of the literature. Indian J Orthop. 2024;58:1548–1556. 10.1007/s43465-024-01237-w PMC11554947 39539337

[3] Dedmond BT , Almekinders LC . Operative versus nonoperative treatment of knee dislocations: a meta‐analysis. Am J Knee Surg. 2001;14(1):33–38. 11216717

[4] Edwards GAD , Sarasin SM , Davies AP . Dislocation of the knee: an epidemic in waiting? J Emerg Med. 2013;44(1):68–71. 22056550 10.1016/j.jemermed.2011.06.064

[5] Everhart JS , Du A , Chalasani R , Kirven JC , Magnussen RA , Flanigan DC . Return to work or sport after multiligament knee injury: a systematic review of 21 studies and 524 patients. Arthroscopy. 2018;34(5):1708–1716. 29429563 10.1016/j.arthro.2017.12.025

[6] Fahlbusch H , Krivec L , Müller S , Reiter A , Frosch KH , Krause M . Arthrofibrosis is a common but poorly defined complication in multiligament knee injuries: a systematic review. Arch Orthop Trauma Surg. 2023;143(8):5117–5132. 10.1007/s00402-022-04730-9 PMC10374851 36520199

[7] Fanelli GC , Stannard JP , Stuart MJ , MacDonald PB , Marx RG , Whelan DB , et al. Management of complex knee ligament injuries. J Bone Joint Surg Am. 2010;92(12):2235–2246. 20844167

[8] Hantes M , Fyllos A , Papageorgiou F , Alexiou K , Antoniou I . Long‐term clinical and radiological outcomes after multiligament knee injury using a delayed ligament reconstruction approach: a single‐center experience. Knee. 2019;26(6):1271–1277. 10.1016/j.knee.2019.08.009 31575512

[9] He J , Geng B , Xu P , Xia Y . Do age and timing influence the outcomes of single‐stage reconstruction of multiple ligament knee injuries? 5‐10 years follow up. Orthop Surg. 2024;16(6):1308–1316. 38644618 10.1111/os.14067 PMC11144514

[10] Held MFG , North D , Von Bormann RB , Wascher DC , Richter DL , Schenck RC . Advances and trends in multiligament injuries of the knee relevant to low‐resource settings. J Arthrosc Surg Sports Med. 2020;1(1):118–125.

[11] Hughes AJ , Li ZI , Garra S , Green JS , Chalem I , Triana J , et al. Clinical and functional outcomes of documented knee dislocation versus multiligamentous knee injury: a comparison of KD3 injuries at mean 6.5 years follow‐up. Am J Sports Med. 2024;52(4):961–967. 38400667 10.1177/03635465241231032

[12] Kanakamedala AC , Sheean AJ , Alaia MJ , Irrgang JJ , Musahl V . Concomitant periarticular fractures predict worse patient‐reported outcomes in multiligament knee injuries: a matched cohort study. Arch Orthop Trauma Surg. 2020;140:1633–1639. 10.1007/s00402-020-03344-3 31980877

[13] Klasan A , Maerz A , Putnis SE , Ernat JJ , Ollier E , Neri T . Outcomes after multiligament knee injury worsen over time: a systematic review and meta‐analysis. Knee Surg Sports Traumatol Arthrosc. 2024;33(4):1281–1298. 39194423 10.1002/ksa.12442 PMC11948183

[14] Levy BA , Dajani KA , Whelan DB , Stannard JP , Fanelli GC , Stuart MJ , et al. Decision making in the multiligament‐injured knee: an evidence‐based systematic review. Arthroscopy. 2009;25(4):430–438. 19341932 10.1016/j.arthro.2009.01.008

[15] Neri T , Myat D , Beach A , Parker DA . Multiligament knee injury. Clin Sports Med. 2019;38(2):235–246. 30878046 10.1016/j.csm.2018.11.010

[16] Ng JWG , Myint Y , Ali FM . Management of multiligament knee injuries. EFORT Open Rev. 2020;5(3):145–155. 10.1302/2058-5241.5.190012 PMC7144894 32296548

[17] Özbek EA , Dadoo S , Grandberg C , Runer A , Cong T , Hughes JD , et al. Early surgery and number of injured ligaments are associated with postoperative stiffness following multi‐ligament knee injury surgery: a systematic review and meta‐analysis. Knee Surg Sports Traumatol Arthrosc. 2023;31(10):4448–4457. 10.1007/s00167-023-07514-9 37486368

[18] Reverté‐Vinaixa MM , García‐Albó E , Blasco‐Casado F , Pujol O , Pijoan BJ , Joshi‐Jubert N , et al. Multiligament knee injuries. Ten years' experience at a public university, level I Trauma Center. Eur J Orthop Surg Traumatol: Orthop Traumatol. 2024;34(3):1349–1356. 10.1007/s00590-023-03807-4 38147073

[19] Richter M , Bosch U , Wippermann B , Hofmann A , Krettek C . Comparison of surgical repair or reconstruction of the cruciate ligaments versus nonsurgical treatment in patients with traumatic knee dislocations. Am J Sports Med. 2002;30(5):718–727. 12239009 10.1177/03635465020300051601

[20] Schenck R . Classification of knee dislocations. Oper Tech Sports Med. 2003;11(3):193–198.

[21] Seroyer ST , Musahl V , Harner CD . Management of the acute knee dislocation: the Pittsburgh experience. Injury. 2008;39(7):710–718. 18472101 10.1016/j.injury.2007.11.022

[22] Tardy N , Boisrenoult P , Teissier P , Steltzlen C , Beaufils P , Pujol N . Clinical outcomes after multiligament injured knees: medial versus lateral reconstructions. Knee Surg Sports Traumatol Arthrosc. 2017;25:524–531. 10.1007/s00167-016-4067-4 27000392

[23] Werner BC , Gwathmey FW , Higgins ST , Hart JM , Miller MD . Ultra‐low velocity knee dislocations: patient characteristics, complications, and outcomes. Am J Sports Med. 2014;42(2):358–363. 24214926 10.1177/0363546513508375

[24] Wilson SM , Mehta N , Do HT , Ghomrawi H , Lyman S , Marx RG . Epidemiology of multiligament knee reconstruction. Clin Orthop Relat Res. 2014:4722603–4722608. 10.1007/s11999-014-3653-3 PMC4117876 24777729

